# Simulation of a Geiger-Mode Imaging LADAR System for Performance Assessment

**DOI:** 10.3390/s130708461

**Published:** 2013-07-03

**Authors:** Seongjoon Kim, Impyeong Lee, Yong Joon Kwon

**Affiliations:** 1 Department of Geoinformatics, University of Seoul, 90 Jeonnong-dong, Dongdaemun-gu, Seoul 130-743, Korea; E-Mail: kimseongjoon@gmail.com; 2 Defense Advanced R&D Center, Agency for Defense Development, Yuseong P.O. Box 35-41, Daejon 350-600, Korea; E-Mail: aubrey@hanmir.com

**Keywords:** ladar, lidar, laser radar, imaging, flash ladar, Geiger mode, FPA, GmAPD, GmFPA, range gated, sensor model, simulation, performance assessment

## Abstract

As LADAR systems applications gradually become more diverse, new types of systems are being developed. When developing new systems, simulation studies are an essential prerequisite. A simulator enables performance predictions and optimal system parameters at the design level, as well as providing sample data for developing and validating application algorithms. The purpose of the study is to propose a method for simulating a Geiger-mode imaging LADAR system. We develop simulation software to assess system performance and generate sample data for the applications. The simulation is based on three aspects of modeling—the geometry, radiometry and detection. The geometric model computes the ranges to the reflection points of the laser pulses. The radiometric model generates the return signals, including the noises. The detection model determines the flight times of the laser pulses based on the nature of the Geiger-mode detector. We generated sample data using the simulator with the system parameters and analyzed the detection performance by comparing the simulated points to the reference points. The proportion of the outliers in the simulated points reached 25.53%, indicating the need for efficient outlier elimination algorithms. In addition, the false alarm rate and dropout rate of the designed system were computed as 1.76% and 1.06%, respectively.

## 1. Introduction

LADAR (Laser Detection and Ranging) calculates target distance ranges by measuring the flight times of the laser pulses transmitted to and reflected from the target surfaces. These ranges can be further converted into a 3D point cloud or a range-image in a local coordinate system by their integration with the position and attitude data acquired from Global Positioning System (GPS)/Integrated Navigation System (INS) sensors mounted with the laser ranging unit.

As an emerging technology, it provides densely sampled 3D points with reliable and consistent quality in an automatic and prompt way. Thus LADAR systems have been widely utilized for various applications in diverse fields. According to their specific applications, various kinds of LADAR systems have been developed with different components and mechanisms (e.g., scanning mechanisms, detector types and sizes, and output data types) [1,2].

In topographic mapping, many applications to derive geospatial information from 3D point clouds have been developed, such as noise reduction [3], classification of ground points [4–6], segmentation of meaningful patches [7–10], Digital Elevation Model (DEM) generation [4,6,11], building reconstruction [12–16], power-line detection [17,18], coastline extraction [19], forest biomass estimation [20–22], and target detection [23–25].

Most systems used in topographic mapping employ a single detector with a scanning system [26]. The detector typically operates in a linear mode, producing an output current linearly proportional to the power of the incident light [27]. By monitoring the output current, the system determines the receiving time of the returned laser pulses using a pulse detection scheme. In addition to the time, recent systems also record the complete waveforms of the returned laser pulses [28]. The waveforms provide additional information about the geometric and physical properties of the targets, particularly those composed of complex objects [28,29]. For example, in forest management, the waveforms are utilized for precise estimations of forest biomass [30–32].

In the defense sector, LADAR with Focal Plane Array (FPA) is more widely used for surveillance and reconnaissance in order to detect obstacles for safety guidance of ground or aerial vehicles [26]. Similar to the CCD of a digital camera, a FPA system, called also “flash LADAR,” can acquire 3D images while retaining the size of the array of detectors with a single laser shot.

For a high sensitivity detector, a Geiger-mode avalanche photodiode (GmAPD) has been recently employed. When the number of incident photons exceeds a predefined threshold, the APD becomes saturated [1,33]. In addition, it outputs only a 1-bit digital state (0 or 1). Geiger-mode avalanche photodiode focal plane arrays (GM-FPAs) have been reported in numerous publications [27,33–37]. GmAPD can provide several benefits [27,34]. Because of the high detection efficiency (up to single-photon sensitivity), it is possible to reduce the laser power for longer ranging distances and system requirements (e.g., size, weight). However, since such highly sensitive detectors inherently suffer from noises, most systems with such detectors employ a range gating scheme to reduce the effect of the noises by limiting the viewing range with a short exposure time [38–40]. Recent advances in CMOS detectors are providing fully integrated scanning LADAR sensors using Geiger mode detectors for automotive applications [41].

As various kinds of LADAR systems have been developed for diverse applications, simulations of such systems have also been studied. Simulation studies are an essential prerequisite for the development of a new LADAR system [42–45]. A simulation can provide: (1) a prediction of the system performance and optimization of the system design; (2) test data to develop and validate the application algorithms dedicated to the given system; and (3) a deeper understanding of LADAR systems for education and training.

Topographic mapping applications have predominantly used airborne LADAR systems, including a laser scanner with a linear mode single detector and a scanning mirror, GPS and IMU. Most simulation studies on such systems have focused on the geometric aspects. For example, the precise modeling of the systematic error of an airborne mapping LADAR system was performed by Schenk [46]. Lohani [47] generated a 3D point cloud for airborne LADAR using geometric modeling. Kukko [48] performed a simulation with the real system parameters of commercial airborne LADAR systems for the analysis of scanning patterns.

The previous studies related to FPA are as follows: the Center for Advanced Imaging LADAR (CAIL) in the University of Utah, USA, performed a modeling simulation for linear mode imaging LADAR to develop LadarSIM, implemented in Matlab [49–51]. A similar work was published by Swedish Defence Research Agency (Totalförsvarets forskningsinstitut, FOI) in Sweden [44]. They developed a modularized computer model, FOI-LadarSIM, which is capable of LADAR simulation. Defence Science and Technology Organisation (DSTO) in Australia developed simulation software of foliage-penetrating LADAR with Matlab, assuming the detector was in Geiger mode [45]. However, there are some limitations when ignoring the noise and the characteristics of GmAPD. Zhao [52], in the National University of Defense Technology (NUDT), China, published a simulation method for imaging laser radar, mainly focusing on the noise model and the related dropouts and outliers. Many researchers have attempted to develop LADAR simulators for their own purposes. Most of them, however, focused on specific scope rather than fully comprehensive aspects.

In this study, we developed a method of comprehensive modeling and simulation for Geiger-mode imaging LADAR with a gate ranging and scanning mechanism. We then predicted and modeled its performance. For high fidelity models, we analyzed previous works and then integrated the rigorous models into a comprehensive method. Our simulator is composed of three main modules: geometry, radiometry, and detection modules. The geometry module defines the rays of laser beams and then determines the locations at which the rays intersect with the target surfaces. The radiometry module computes the powers of the return pulses and generates the waveforms. The detection module finally generates the time when each pixels in a detector perceives the first photon. Using the proposed simulation of three modules, the reference data, as well as the corresponding simulated point cloud, are generated. Finally, we evaluated the sensor performance based on the simulation by comparing the simulated points with the reference points.

This research reliably verifies the data from a new type of LADAR system with given parameters and assesses its performance using indicators, such as the amount of noise and false alarms in advance of developing hardware. Our simulator also provides a diversity of simulated data for the development of application algorithms that should be optimized for a real system.

The paper is organized as follows: Section 2 describes the modeling principles and simulation processes. Section 3 presents the experimental results with the implemented simulator and our analysis of the performance assessment with the given system parameters. Finally, we present our conclusions and future research directions.

## 2. LADAR Modeling and Simulation

**Figure 1.**
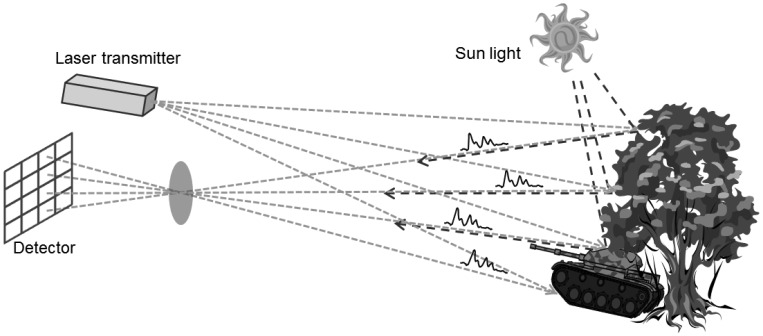
Principle of a LADAR sensor. The transmitter emits pulsed laser repetitively with a constant rate. The laser pulses are then backscattered on the surfaces of targets or background. Each pixel of the detector measures the travel time of the laser pulse by collecting the return pulse. The collected incident energy also includes noises such as sunlight and dark count due to thermal effects.

LADAR (or laser radar) generates 3D point cloud and range images by measuring the flight times of laser pulses. Figure 1 illustrates how the system acquires the point cloud. First, a laser pulse is transmitted to the surfaces of the targets and background. The pulse is then backscattered after interacting with the surfaces. The reflected pulse energy passes through the optics and reaches the receiver. A detector at the receiver senses the incident energy. A Geiger-mode detector subtly responds to the first incident photon and is saturated regardless of the amount of received energy. In this way, it provides the time at which the first photon is perceived, while the linear mode detector generates the waveform of the return pulse.

Ideally, a detector senses only the pulse energy emitted by the transmitter. However, internal and external noise energies are also detected by the receiver along with the return pulse. The main causes of noise are the backscattered solar radiation and dark count due to thermal effects.

**Figure 2.**
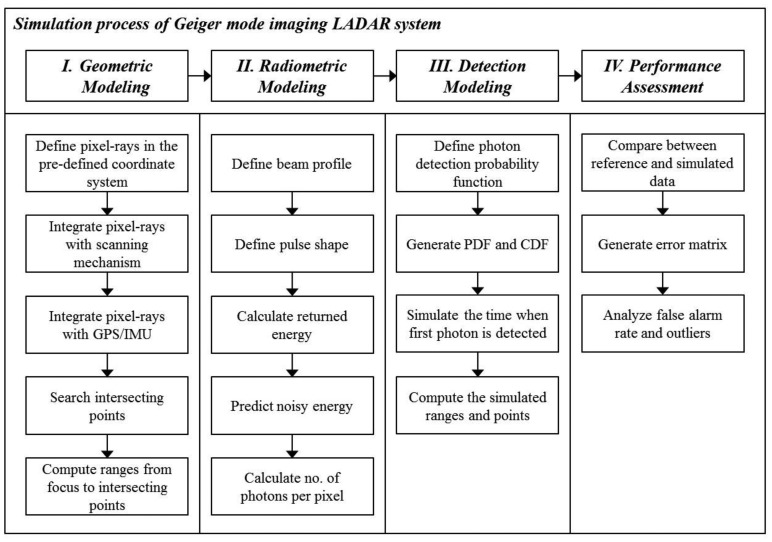
Simulation process of Geiger-mode imaging LADAR. Geometric model finds where the information of each pixel comes from. Radiometric model computes how much energy strikes the pixels, including noise energies. Detection model generates the simulated time when the first photon is detected based on a probability function. Simulated point cloud was compared point by point to the reference data generated in the geometric simulation.

For the simulation of a LADAR system, three models were required (Figure 2). The first process, based on the geometric model, finds where the information of each pixel comes from. This process can be executed by establishing the geometric relationships between the pixels in the detector and target surfaces. This enables the computation of 3D points as the intersection points between the ray passing from each pixel to a focal point and its corresponding surface. They are not affected by the radiometric conditions or the nature of the detector. Therefore, this point cloud can be used as a reference for the simulation outputs of the radiometric and detector models.

In the second step, the radiometric model computes how much energy strikes the pixels, including noise energies. First, the transmitted energy of each pixel is calculated using the predefined beam profile. The return energy is computed using the laser equation with the radiometric and optical parameters and ranges calculated from the geometric model. Using the radiometric model, we can compute the number of incident photons according to time.

The detection model generates the simulated time when the first photon is detected based on a probability function. It includes the effect of APD timing jitter—statistical time interval between the pulse arrival and the signal output of APD. But afterpulsing effect, causing the noise, is not considered in this paper. According to earlier research, the saturation of a Geiger-mode detector from all light sources follows Poisson statistics under several assumptions [1]. The point cloud generated using the detection model includes outliers. For a performance assessment, the simulated point cloud was compared point by point to the reference data generated in the geometric simulation. In general, it is difficult to compare two point cloud sets, since the correspondences between the individual points in the two sets are difficult to establish. We were able to identify every corresponding point of the reference and the simulated data; therefore, it was possible to compare point by point. Using the error matrix of the compared results, we computed the false alarm rate, dropout rate and outlier ratio of the simulated point cloud.

### 2.1. Geometric Modeling

The purpose of the geometric modeling is to identify the source of each pixel's information, or the point at which the transmitted laser pulse is reflected on a target surface. To find this point, the geometry of the laser pulse needs to be determined, both the direction and origin. Geometric modeling can then establish the ray model of the laser pulse and compute the intersection point. We can determine the range from the origin to the intersection point and the reflectance of the intersected surface, which are used for the radiometric modeling described in Section 2.2.

The ray model can be defined by the geometric integration of the sub-modules in the LADAR system. The sub-modules are a GPS/INS and a scanning mechanism, each of which has its own coordinate system. Therefore, they should be redefined in a common coordinate system using a geometric transformation based on the geometric relationships between the sub-modules.

#### 2.1.1. Detector

**Figure 3.**
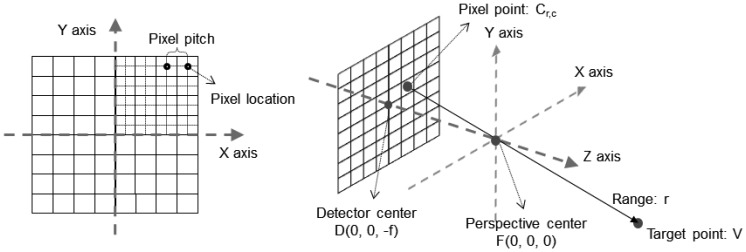
Sensor coordinate system and a pixel ray model. The left one is 2D view of the detector and the right figure shows 3D view. Subscript (*r*, *c*) are the row and column indices respectively.

An FPA detector system has N × N pixels. The acquired information for each pixel originates from the target point on the ray passing the pixel and the perspective center. The pixel location, the perspective center and the target point are collinear. The line equation of the pixel ray can be established with three points. To define the ray, we defined the sensor coordinate system of the detector as shown in Figure 3. Based on the sensor coordinate systems, we defined the line equation as Equation (1). The target point *V* is represented with the origin (perspective center) *F*, the direction *u*_0_, and the range *r* from the principal point to the target point. Direction *u*_0_ is a unit vector from the location of pixel *C*_*r*,*c*_ to the origin *F*. Subscripts (*r*, *c*) are the row and column indices respectively.

(1) $$V=u_{0}\cdot r+F=\frac{\vec{C_{r,c}F}}{\left\|\vec{C_{r,c}F}\right\|}.r$$

**Figure 4.**
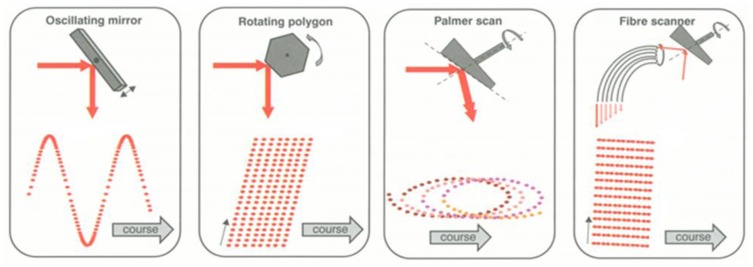
Scanning mechanisms and resulting ground patterns [2].

**Figure 5.**
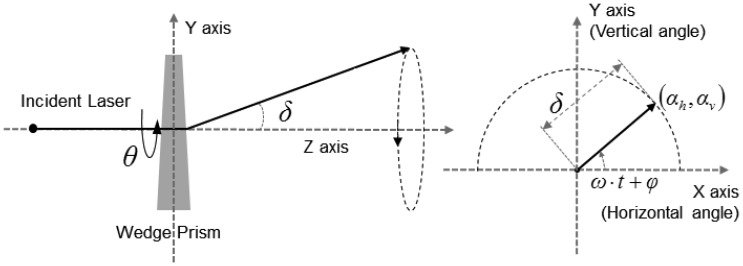
Geometric deflection by a wedge prism. The horizontal and vertical angular positions (*α_h_*, *α_v_*) created by a set of four prisms for a zigzag pattern can be expressed by a trigonometric function. And 3D transformation matrices for steering the pixel ray horizontally and vertically can be established.

**Figure 6.**
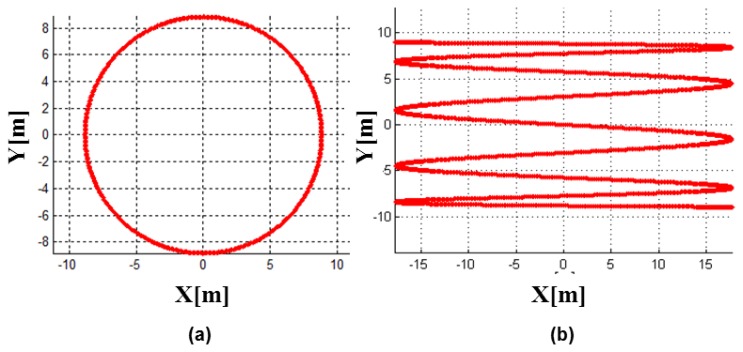
Scan pattern by the wedge prisms with deflection angle of 0.256° at 1 km (**a**) and pattern using two pairs of counter-rotating optical wedge prisms with the horizontal deflection angle of 0.506° and the vertical deflection angle of 0.506° at 1 km (**b**). Combination of two wedge prisms makes it possible to implement the reciprocating pattern, which is parallel to the vertical or horizontal axis.

A LADAR system employs the scanning mechanism to increase its coverage. There are a variety of scanning mechanisms and each has its own scanning pattern (Figure 4). Each LADAR system adopts a scanning mechanism suited to its own purpose, considering the strengths and weaknesses of each [2]. We performed modeling of the zigzag scanning pattern using Risley prisms with two pairs of counter-rotating optical wedge prisms. A wedge prism is a prism with a shallow angle between its input and output surfaces, and a pair of wedge prisms is called Risley prism pair. A Risley prism scanner can be developed so as to be relatively compact with low-power operation [53]. As shown in the left of Figure 5, a wedge prism can steer the laser beam with a deflection angle *δ* and a circle pattern by rotating a lens (the left of Figure 6). Furthermore, the combination of two wedge prisms makes it possible to implement the reciprocating pattern, which is parallel to the vertical or horizontal axis. In addition, a zigzag pattern is feasible with two Risley prisms, as shown in the right of Figure 6.

(2) $$\begin{array}{ll} \alpha_{h}=\sum_{k=1}^{4}\delta_{k}\cdot \cos(\omega_{k}t+\varphi_{k}) & \alpha_{v}=\sum_{k=1}^{4}\delta_{k}\cdot \sin(\omega_{k}t+\varphi_{k}) \end{array}$$

(3) $$\begin{array}{ll} R_{h}=\left[\begin{array}{ccc} \cos(\alpha_{h}) & 0 & \sin(\alpha_{h})\\ 0 & 1 & 0\\ -\sin(\alpha_{h}) & 0 & \cos(\alpha_{h}) \end{array}\right] & R_{v}=\left[\begin{array}{ccc} 1 & 0 & 0\\ 0 & \cos(\alpha_{v}) & -\sin(\alpha_{v})\\ 0 & \sin(\alpha_{v}) & \cos(\alpha_{v}) \end{array}\right] \end{array}$$

(4) $$R_{0}^{L}=R_{h}\cdot R_{v}=\left[\begin{array}{ccc} \cos(\alpha_{h}) & \sin(\alpha_{h})\cdot \sin(\alpha_{v}) & \sin(\alpha_{h})\cdot \cos(\alpha_{v})\\ 0 & \cos(\alpha_{v}) & -\sin(\alpha_{v})\\ -\sin(\alpha_{h}) & \cos(\alpha_{h})\cdot \sin(\alpha_{v}) & \cos(\alpha_{h})\cdot \cos(\alpha_{v}) \end{array}\right]$$

The horizontal and vertical angular positions (*α_h_*, *α_v_*) created by a set of four prisms for a zigzag pattern can be expressed by a trigonometric function with a rotational speed *ω*, phase delay *φ*, time *t* and deflection angle *δ* as in Equation (2). The 3D transformation matrices for steering the pixel ray horizontally and vertically are shown in Equation (3), and Equation (4) is the 3D transformation matrix $R_{0}^{L}$ for the zigzag scan pattern. We assumed that the pixel rays deflect at the principal point in the sensor coordinate system.

#### 2.1.2. Geometric Transformation

**Figure 7.**
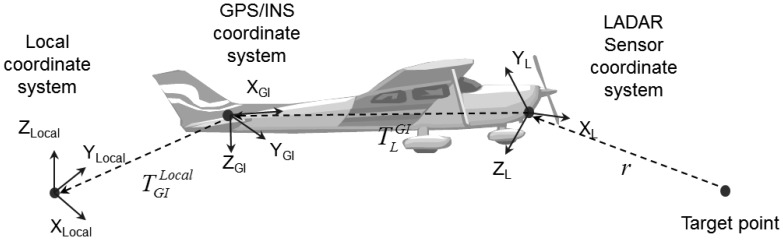
Geometric relationships among sub-modules [46]. The aim is to geometrically transform the vector from the origin to target point represented in LADAR sensor coordinate to the vector in the local coordinate system.

We then transformed the line-equation in Equation (1) into a local coordinate system. Usually, GPS/INS and laser scanners are mounted on a platform together. GPS/INS provides the position and the attitude of the local coordinate system. Figure 7 shows the geometric relationships of a LADAR system. In Figure 7, $T_{GI}^{\text{Local}}$, which is represented in the local coordinate system, is the position of GPS/INS. $T_{L}^{GI}$ is the offset between the GPS/INS and laser scanner. Based on this geometric relationship, Equation (5) was derived, where *R* indicates a rotational matrix for the geometric transformation, and *T* is a translation vector between the origins of the coordinate systems. $R_{L}^{GI}$ is the rotational matrix from the sensor coordinate system of the laser scanner to the GPS/INS coordinate system, and $R_{GI}^{\text{Local}}$ is the rotational matrix from the GPS/INS coordinate system to the local coordinate system.

$$V_{\text{Local}}=R_{GI}^{\text{Local}}(R_{L}^{GL}\cdot R_{0}^{L}\cdot u_{0}\cdot r+T_{L}^{GI})+T_{GI}^{\text{Local}}$$

#### 2.1.3. Geometric Errors

All sub-modules in LADAR systems, such as GPS/INS and laser scanners, possess some systematic and random errors. There are two kinds of errors in LADAR systems. The first comprises the individual sensor errors, and the second the integration errors [46]. The former is inherently caused by the sensors themselves. Integration errors stem from the geometric integration among sensors. The integration errors in the LADAR system occur predominantly from measurement errors associated with the mounting parameters and bore-sight angles. In this study, we identified the significant error factors and took them into consideration.

#### 2.1.4. Input Models and Ray-Tracing

The direction and origin of the pixel rays can be represented as follows. The true range of the pixel ray can be calculated by searching the intersecting surface. However, a real LADAR system handles tens of thousands of laser pulses per second. Furthermore, LADAR simulation executes a tremendous number of geometric operations to search for the intersecting points between the pixel rays and the target surfaces [54]. We employed a ray-tracing algorithm to rapidly process the overloaded geometric computations. For this, we used the B-rep (Boundary representation) structure, which is a method for representing shapes as a set of facets, for the input data, such as the target and the background model, because of some advantages that we will discuss in Section 3.2.

**Figure 8.**
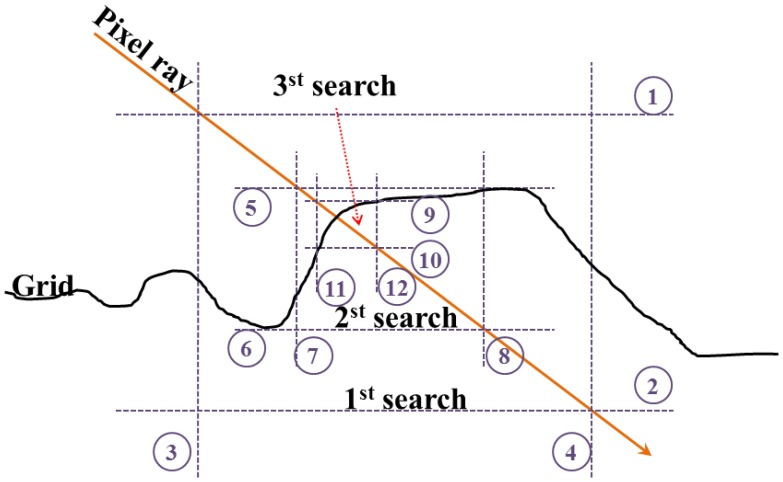
Concept of the ray-tracing algorithm [55]. Ray-tracing algorithm used in the simulation searched the intersecting cell by recursively reducing the vertical and horizontal range until stability was achieved.

The particular details of the ray-tracing used are as follows [55]. First, a grid structure was generated, and all of the facets were linked to their corresponding cells in the grid, according to the horizontal locations. Each cell in the grid has maximum and minimum height values calculated from the boundary point of the linked facets. The aim of ray-tracing is to find the cell with candidate facets that have the highest possibility of intersecting the pixel ray. The ray-tracing algorithm used in the simulation searched the intersecting cell by recursively reducing the vertical and horizontal range until stability was achieved (Figure 8).

### 2.2. Radiometric Modeling

The purpose of radiometric modeling is to calculate the number of incident photons that enter the detector pixels. The radiometric model uses the range computed in the geometric simulation, the radiometric and optical parameters of the system.

Ideally, the photons that strike the pixels of the detector are from the laser energy emitted from the transmitter. However, the detector collects not only the reflected laser energy, but also the energy caused from the backscattered solar radiation. Furthermore, the dark count can also cause false alarms. The radiometric model deals with the reflected pulse energy and these noise sources [1,42,56].

#### 2.2.1. Laser Beam Model

**Figure 9.**
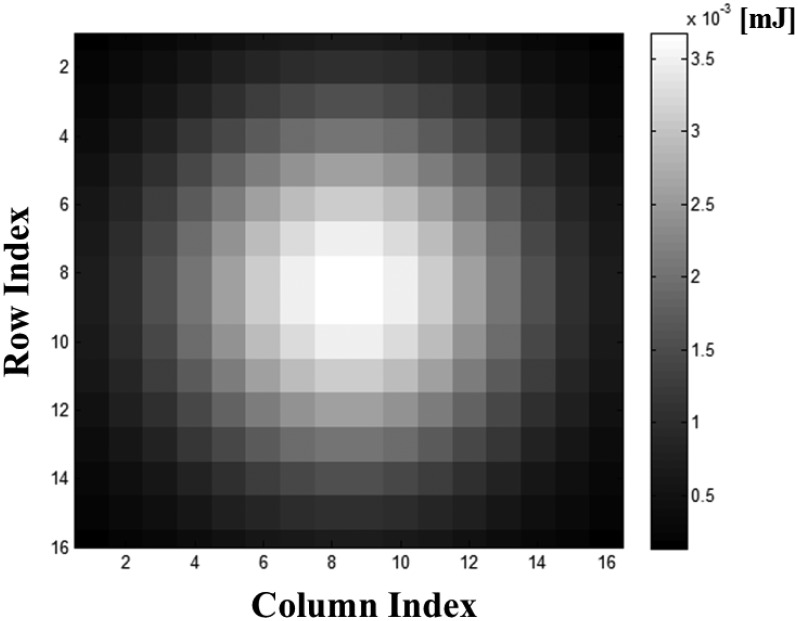
Intensity distribution of a simulated Gaussian beam profile with the array of 16 × 16, pulse energy of 0.4 mJ and 4.8 mrad beam divergence. Normalizing by the sum of Gaussian model and multiplying the pulse energy, we considered the energy loss due to the difference between array size of detector and beam width.

The intensity of the laser beam across the range is not uniform, but varies in the spatial and temporal domains [42]. The intensity of the beam is not uniform across the range from the center axis, which is the direction of the beam. It is defined as a beam profile and depends on the shape of the emitter and the technique used to generate the laser light. Figure 9 shows an example of Gaussian beam profile. The irradiance is expressed as follows [42]:

(6) $$I(d)=I_{0}\cdot e^{-2{\left(\frac{d}{BW}\right)}^{2}}$$

where *d* is the distance measured from the central axis of the beam in the cross-section; *I*_0_ is the maximum irradiance of the beam; and *Bw* is the beam half-width. Commonly, the irradiance is about 14% (*I*_0_/*e*^2^) at *d* = *Bw*.

**Figure 10.**
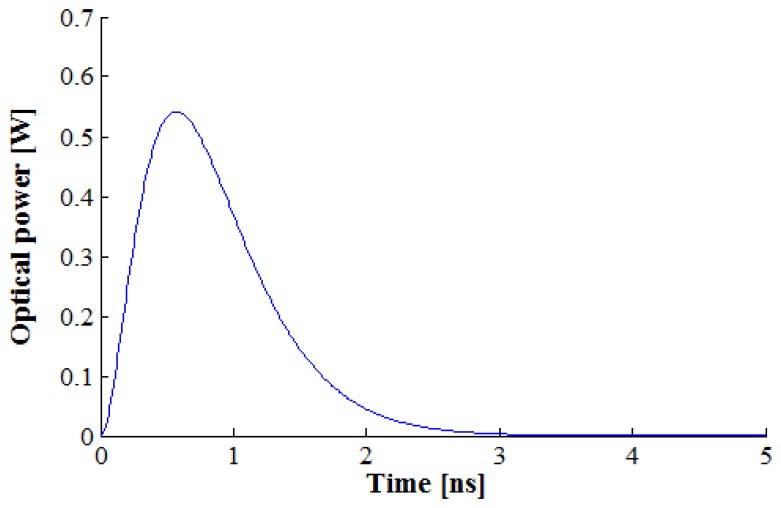
Pulse model used in this study.

In the temporal domain, the laser signal is modeled as a pulse. There are several pulse models with different shapes. The pulse model used in this study was suggested in [42]. It is represented in Figure 10 and expressed as follows:

(7) $$\begin{array}{ll} p(t)={\left(\frac{t}{\tau }\right)}^{2}\cdot e^{-\frac{t}{\tau }}, & \tau =\frac{\text{FWHM}}{3.5} \end{array}$$

where *FWHM* is the full width at half of the maximum of the pulse.

#### 2.2.2. Returned Energy Calculation

The returned laser energy is calculated using a LADAR range equation [56]. Assuming that, for an extended target, the footprint of the beam is smaller than the target surface, the returned power can be calculated using the transmitted power *P_t_*, the travel distance of the laser beam *r*, the reflectance of the target surface *ρ*, and the aperture diameter of the receiver *D*, as represented in Equation (8). In that equation, Ω*_s_* is the scattering steradian solid angle of the target. For Lambertian targets (diffuse targets), Ω*_s_* is replaced by the solid angle of *π* steradians. *η_sys_* and *η_atm_* are the efficiency values of the optics of the system and the atmospheric attenuation, respectively. These variables can be written as Equations (9) and (10). The round trip laser pulse, *η_atm_*, is the square of the atmospheric attenuation in Equation (9); and *η_sys_* can be represented as the product of the fill factor *T_FF_*, the bandpass filter transmittance *T_BPF_*, the ND (Neutral Density) filter transmittance *T_ND_*, transmitter optics transmittance *T_T_* and receiver optics transmittance *T_R_*. With the previous assumption, substituting Equations (9) and (10) into Equation (8) leads to Equation (11):

(8) $$P_{r}=P_{t}\cdot \rho \cdot \frac{1}{\Omega_{s}R^{2}}\cdot \frac{\pi D^{2}}{4}\cdot \eta_{\text{atm}}\cdot \eta_{\text{sys}}$$

(9) $$\eta_{\text{atm}}=T_{\text{atm}\_\text{trancemitted}}\cdot T_{\text{atm}\_\text{received}}=T_{\text{atm}}^{2}$$

(10) $$\eta_{\text{sys}}=T_{\text{BPF}}\cdot T_{ND}\cdot T_{FF}\cdot T_{T}\cdot T_{R}$$

(11) $$P_{r}=\frac{P_{t}\cdot \rho \cdot D^{2}\cdot T_{\text{atm}}^{2}\cdot T_{\text{BPF}}\cdot T_{ND}\cdot T_{FF}\cdot T_{T}\cdot T_{R}}{4R^{2}}$$

#### 2.2.3. Noise Energy Calculation

The main sources of the noises occurring in the detector are reflected sunlight and dark count. They contribute to false alarms by arriving at the detector before the returned laser pulse. The sunlight (solar radiation) is collected by the receiver, although it does not originate from the transmitter. The incident energy of the backscattered solar radiation is given in Equation (12), where *E_si_* is the solar irradiance in a unit of W/m^2^/nm; *δ_λ_* is the electromagnetic bandwidth in of the bandpass filter; *δ_t_* is the unit sampled time bin (the temporal resolution) of the system clock that measures the time; *A* is the area covered within the *IFOV* (instantaneous field of view) in a unit of m^2^ and is calculated as in Equation (13). Equation (15) can be derived from the substitutions of Equation (10), (13) and (14) into Equation (12):

(12) $$E^{\text{solar}}=E_{si}\cdot \delta_{\lambda }\cdot \delta_{t}\cdot A\cdot \rho \cdot \frac{1}{\Omega_{s}R^{2}}\cdot \frac{\pi D^{2}}{4}\cdot \eta_{\text{atm}}\cdot \eta_{\text{sys}}$$

(13) $$A={\left(R\cdot \text{IFOV}\right)}^{2}$$

(14) $$\eta_{\text{atm}}=T_{\text{atm}\_\text{received}}=T_{\text{atm}}$$

(15) $$E^{\text{solar}}=\frac{E_{si}\cdot \delta_{\lambda }\cdot \delta_{t}\cdot \rho \cdot {\text{IFOV}}^{2}\cdot D^{2}\cdot T_{\text{atm}}\cdot T_{\text{BPF}}\cdot T_{ND}\cdot T_{FF}\cdot T_{R}}{4}$$

The expected number of photoelectrons created by the dark count due to the thermal effects within the detector is determined using Equation (16), where *f_dc_* is the dark count rate in a unit of Hz, although the dark count does not actually generate photoelectrons [1,33,37]. This assumes that the dark count is uniformly distributed in the time domain, and that every pixel in the detector has the same dark count:

(16) $$E[N^{dc}]=f_{dc}\cdot \delta_{t}$$

#### 2.2.4. Photons Per Pixel

FPA imaging systems have a detector consisting of an N × N arrayed pixel. The simulation of an imaging system requires the computation of the incident energy for each pixel, including the noise. For this, we derived an equation to compute the incident energy for each pixel under the following assumptions. The first assumption is that N × N laser beams, which we call sub-beams, are independently transmitted from the arrayed pixels and return to the pixel after reflecting off the target surfaces. The other is that the incident noises of each pixel are the same. Under these assumptions, we can calculate the incident energy per pixel.

The transmitted energy of the pulse *E_pulse_* can be determined with the average power of the laser beam *P_t_* and the repetition rate of the pulse *f_pulse_*, as show in Equation (17). Then, the pulse energy is divided into each pixel according to the beam profile. Based on the Gaussian profile as in Equation (6), the energy of the pixel located at (*r*, *c*), *E*_*r*,*c*_, can be represented as Equation (18). The function *Nor* indicates the normalization of the beam profile to produce a summation.

The returned energy of the sub-beam collected by a pixel can be derived using Equation (19), where $E_{r,c}^{\text{laser}}$ is the total energy received by (*r*, *c*) the pixel. Then the return energy of the pixel $E_{r,c}^{\text{laser}}$ is modeled in the time domain with the pulse model as Equation (20). We defined the time bin *t* as an element of set $T(t\in T\;\text{and}\;T=\left\{k\cdot \delta_{t}+T_{\min}|k\;\text{is an integer satisfying}\;0\leq k\leq \frac{T_{\max}-T_{\min}}{\delta_{t}}\}\right)$ in the range gate. The range gate is a length of measurement in the time domain. *t_r_* is the converted time from the round-trip distance between pixel and reflected point, and it is for shifting the pulse model by the delayed time. The received energy detected by the (*r*, *c*) pixel at a certain time *t* follows Equation (20). Finally, the expected number of the photons collected by the pixel can be determined by dividing the energy of the unit photoelectron, as in Equation (21), where *h* is Planck's constant and *v* is the frequency of the laser light. The expected number of photons from the solar radiation is expressed with the speed of the light *c* and the wavelength *λ*, as in Equation (22):

(17) $$E_{\text{pulse}}=\frac{P_{t}}{f_{\text{pulse}}}$$

(18) $$E_{r,c}=\text{Nor}\left[e^{-2{\left(\frac{\text{dist}(r,c)}{Bw}\right)}^{2}}\right]\cdot E_{\text{pulse}}$$

(19) $$E_{r,c}^{\text{laser}}=\frac{E_{r,c}\cdot \rho \cdot D^{2}\cdot T_{\text{atm}}^{2}\cdot T_{\text{BPF}}\cdot T_{\text{PLF}}\cdot T_{NA}\cdot T_{FF}}{4R^{2}}$$

(20) $$E_{r,c,t}^{\text{laser}}=\text{Nor}\left[{\left(\frac{t-t_{r}}{\tau }\right)}^{2}\cdot e^{-\frac{t-t_{r}}{\tau }}\right]\cdot E_{r,c}^{\text{laser}}$$

(21) $$E[N_{r,c,t}^{\text{laser}}]=\frac{E_{r,c,t}^{\text{laser}}}{h\cdot v}=\frac{E_{r,c,t}^{\text{laser}}\cdot \lambda }{h\cdot c}$$

Consequently, the expected number of photoelectrons sensed by the (*r*, *c*) pixel at a certain time can be calculated using Equation (22), where *E*[*N^dc^*] is not affected by *PDE* (photon detection efficiency), because the dark count occurs in the APD circuit:

(22) $$E[N_{r,c,t}]=\text{PDE}\cdot (E[N_{r,c,t}^{\text{laser}}]+E[N^{\text{solar}}])+E[N^{dc}]$$

### 2.3. Detection Modeling

#### 2.3.1. Detection Probability

The detection simulation determines the simulated time when each pixel detects the first photon. A Geiger mode detector can only perceive the primary photon, because it takes a few microseconds to recover from the saturation by the photon. The saturation of the detector by the laser pulse and noise follows Poisson statistics [1,27].

In a certain time interval (time bin), the probability *P*(*m*) that a pixel detects a number of photons is determined using Equation (23) [1,33,37], where *λ* is the expected number of incident photons. By substituting *λ* with Equation (22), the number of photons sensed by the pixel in the time bin *t*, *P*(*m*; *t*), is derived as Equation (24). Since a Geiger mode detector is saturated if at least one photon is sensed, the complementary event occurs when no photon is sensed (*m*= 0). The probability that at least one photon is detected can be expressed as Equation (25) [1]:

(23) $$P(m)=\frac{1}{m!}\cdot \lambda^{m}\cdot e^{-\lambda }$$

(24) $$P(m;t)=\frac{1}{m!}\cdot {\left(E[N_{r,c,t}]\right)}^{m}\cdot e^{-E[N_{r,c,t}]}$$

(25) $$P(t)=1-e^{-E[N_{r,c,t}]}$$

#### 2.3.2. Detection Process

**Figure 11.**
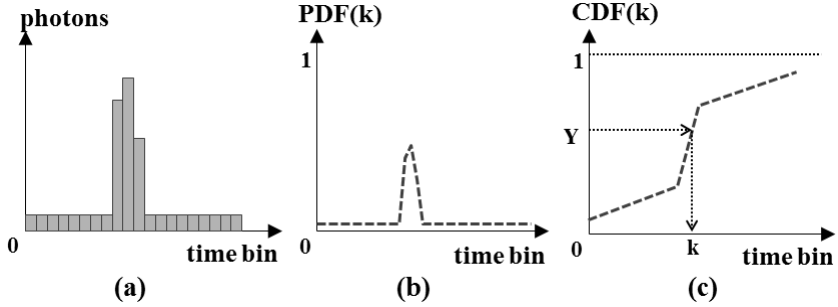
Simulation of the time at which the pixel detects the primary photon ((**a**) photons; (**b**) probability density function; (**c**) cumulative distribution function).

Using the detection probability of one pixel at each time bin, we can generate the simulated time when each pixel detects the photons as follows [1]:

(1) Compute the incident photons for each time bin within the range gate using Equation (23), as shown in Figure 11(a). These include the expected number of photons created by the transmitted laser pulse, the backscattered solar radiation and the dark count.
(2) By computing the probabilities that the pixel detects at least one photon for each time bin using Equation (25), generate a PDF (Probability Density Function) as shown in Figure 11(b),
(3) Convert the PDF into a CDF (Cumulative Distribution Function) using Equation (26), as illustrated in Figure 11(c).
  (26) $$\text{CDF}(k)=\sum_{i=1}^{k}\text{PDF}(t_{i})$$
(4) Generate a random number Y from 0 to 1 that follows the uniform distribution. Then, search for the bin *k* that satisfies Y = *CDF*(k). The bin *k* is the simulated time when the pixel detects the primary photon.

## 3. Experiments

We performed an experiment to verify the proposed methods for LADAR simulation. Based on the simulation results, we also assessed the performance of the LADAR system with the designed system parameters and mission scenario.

### 3.1. Simulator Development

**Figure 12.**
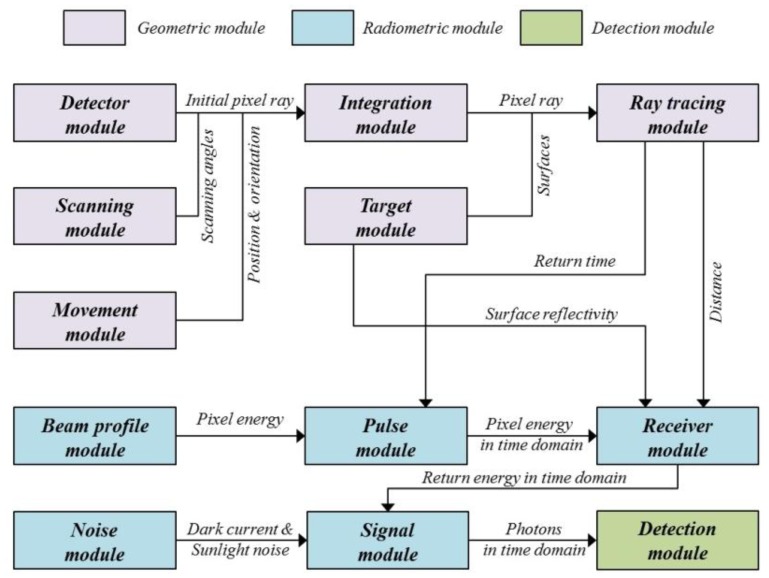
Main modules in the LADAR simulation program and their relationships. Geometric module outputs the range from the perspective center to the intersection point. Radiometric module computes the incident energy of each pixel from both the transmitted laser pulse and the noise and generates the number of incident photons on each pixel per time bin. Detection module calculated the simulated time at which the pixel perceives the incident photons based on the probability model.

The simulation program was implemented using C++ language. The simulator is mainly composed of three parts: geometry, radiometry, and detection, as shown in Figure 12. A geometric module identifies the source of the information of each pixel in the detector based on the geometric relationships between the LADAR system and the target [55]. It outputs the range from the perspective center to the intersection point. The radiometric module computes the incident energy of each pixel from both the transmitted laser pulse and the noise and generates the number of incident photons on each pixel per time bin. The detection module calculated the simulated time at which the pixel perceives the incident photons based on the probability model. Table 1 shows the modules in detail.

### 3.2. Input Data

The developed simulator employs 3D polyhedral models expressed in B-rep. As the input data of the LADAR simulator, B-rep models retain some advantages. They simplify the geometric operations in simulations without the need for interpolation. Moreover, they are very flexible in varying the given system parameters according to the mission scenario. For example, if the simulator uses range images as the input data instead of the B-rep models, many different images are needed to account for the various positions and orientations of the sensors based on the given mission scenario.

**Figure 13.**
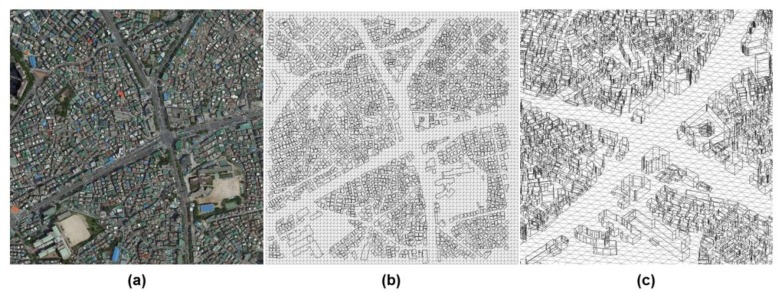
City model of the test area. The test area is a part of Yeongdeungpo-gu in Seoul, South Korea ((**a**) image; (**b**) 2D view of city model; (**c**) 3D zoomed-in view).

Figure 13 represents the 3D city model that was generated for the simulation experiments. The city model is a part of Yeongdeungpo-gu in Seoul, South Korea. It was generated by combining the horizontal boundaries from digital maps and the corresponding height information from airborne LADAR data. It uses a total of 32,968 polygons to represent the ground and buildings.

### 3.3. System Parameters

A LADAR system has three sub-modules, such as a GPS/INS and laser scanner. The laser scanner consists of various components, such as a laser transmitter, optics, a receiver, a detector, and a scanning device. Their system parameters need to be determined for each simulation. Tables 2, 3, 4 and 5 describe the main parameters and the values for each component. In Table 2, the laser mean power is the energy emitted by the transmitter per second. Because 25,000 laser pulses were transmitted, the energy of the laser pulse was 0.4 mJ; thus, its peak power was 400 kW, which is the pulse energy divided by the pulse width of 1 ns.

Table 3 describes the parameters related to the scanning mechanism. The array size of the detector adopted in the LADAR system is small; thus, it is necessary to employ a scanning mechanism to enlarge the coverage. As seen in Table 2, Lenses 1 and 2 had the same deflection angle and phase angle, but they rotated in opposite directions. Lenses 3 and 4 also retained the same properties. The phase angle determines the direction of deflection for a transmitted laser beam. Lenses 1 and 2 enable a horizontal reciprocating motion for the laser beam. Lenses 3 and 4 determine the vertical motion.

Table 4 shows the information about the detector. In order to simulate realistic and precise waveforms of a pixel, we divided a pixel into 6 × 6 sub-pixels to consider multiple echoes. Each sub-pixel received an echo. Assuming an echo is originated from the individual reflected sub-pixel beam, each sub-pixel beam is separately processed in geometric and radiometric simulation. And the waveform of a pixel is generated by summing the echoes of sub-pixel beams.

The dark count is the noise generated on the circuit board due to thermal activity. The occurrence rate was 20 kHz, which is the average number of saturation counts per second even in complete darkness.

Most of the parameters listed in Table 5 are associated with the energy efficiency when the return pulses pass through the optical devices. The bandpass filter permits the incident light of a specific wavelength to pass, and bandpass width is the range of the wavelength. The transmitter and receiver efficiencies are the attenuations due to other optical devices, such as lenses and prisms. Solar irradiance is the measured amount of sunlight striking a square meter of the Earth's atmosphere or surface. It is depending on many factors such as the position of the sun, the weather condition, the season and so on. In this experiment, we used the solar irradiance of 0.3 W/m^2^/nm approximately, which is the value corresponding to 1,560 nm wavelength of the laser in the solar radiance spectrum curve for direct light at sea level [57]. Selection of laser wavelength depends on the application of the sensor. For example, most airborne topographic mapping LADAR systems use 1,064 nm diode pumped YAG lasers. Bathymetric systems generally use 532 nm lasers that can penetrate water with less attenuation [28]. In this study, we focused on the sensors for military application, where 1,560 nm or 1,550 nm lasers are usually preferred, because it is eye-safe at much higher power levels for longer range measurments [26].

### 3.4. Platform Parameters

**Figure 14.**
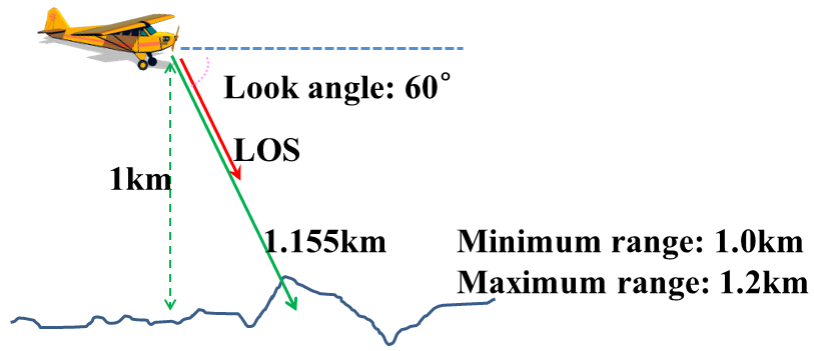
Scenario of the LADAR system for simulation. The aerial platform is assumed to be in the midair at 1 km with the speed of 0 m/s. Besides, the LADAR sensor is mounted obliquely to look targets and background slantingly.

Figure 14 illustrates the position and attitude of the platform mounted with the LADAR system under simulation. The location of the platform was (100, -400, 1000) m in the local coordinate system, and the look angle between the horizon and the LOS (Line of Sight) of the LADAR sensor was 60 degrees. Thus, the distance between the sensor and the target was about 1.2 km, and we determined the measuring range to be 0.2 km (from 1.0 km to 1.2 km). The LADAR system acquired the point cloud for 0.1 s.

### 3.5. Results and Discussion

**Figure 15.**
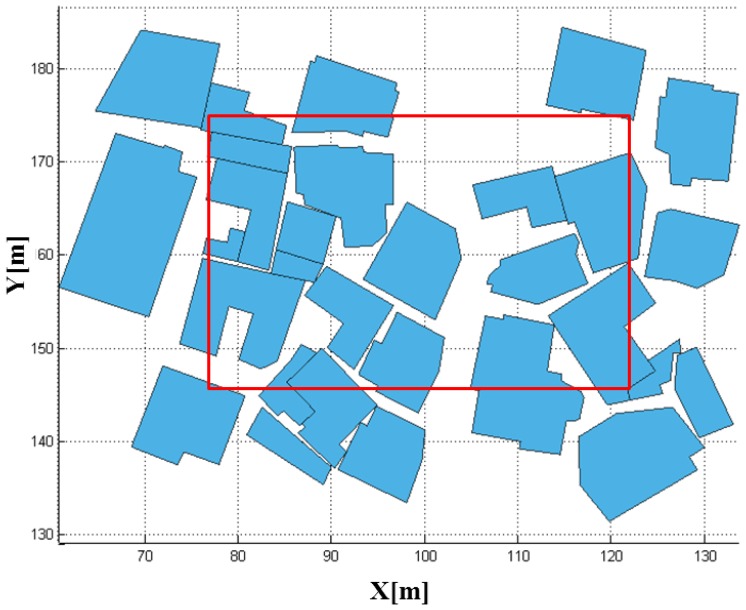
Target models (buildings) in the test area (within red boundary).

**Figure 16.**
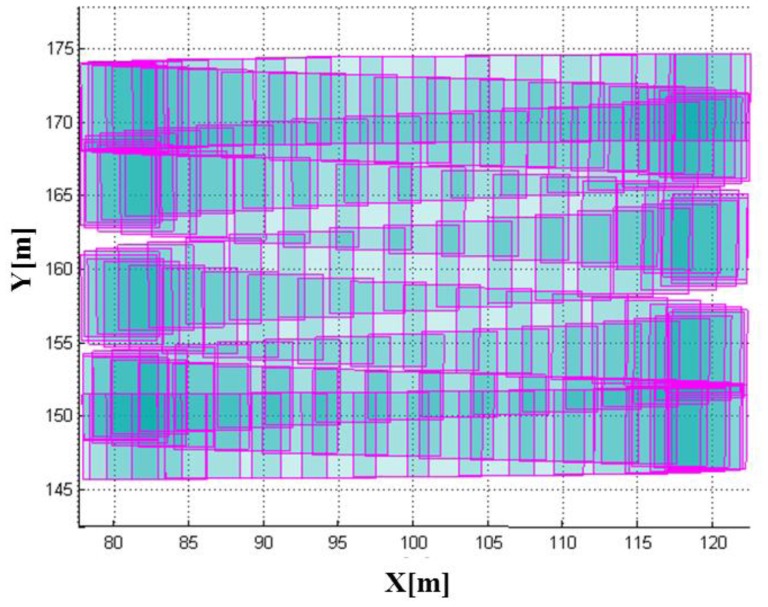
Coverage of FPAs by the scanning mechanism is the one bounded with red box in Figure 15.

**Figure 17.**
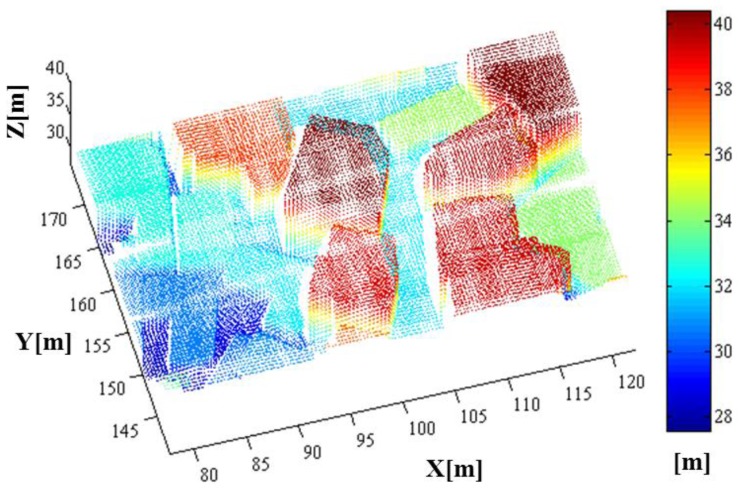
Reference point cloud generated by geometric simulation. They are not affected by the radiometric conditions or the nature of the detector. Therefore, it can be used as a reference for the simulation outputs of the radiometric and detector models.

Having the system and platform parameters established, we were able to perform the LADAR simulation. The coverage of the simulated data with these parameters overlapped with the target models in Figure 15. Figure 16 shows the coverage of the FPAs at each laser shot. Figure 17 represents the point cloud generated by the geometric simulation—the computation of the intersection points between the rays of the sub-beams and the surfaces. Because of computing the intersecting point, all of points are located on their intersecting surfaces, and there is no outlier (noise). Geometric simulation does not consider the radiometric and electronic (photon detection) aspect—such as characteristics of laser, attenuations, beam interaction, noise and detector. Therefore, the point cloud generated in geometric simulation is true of the point cloud resulting from radiometric and detection simulation. This reference point cloud will be used to assess the detection performance in following section.

**Figure 18.**
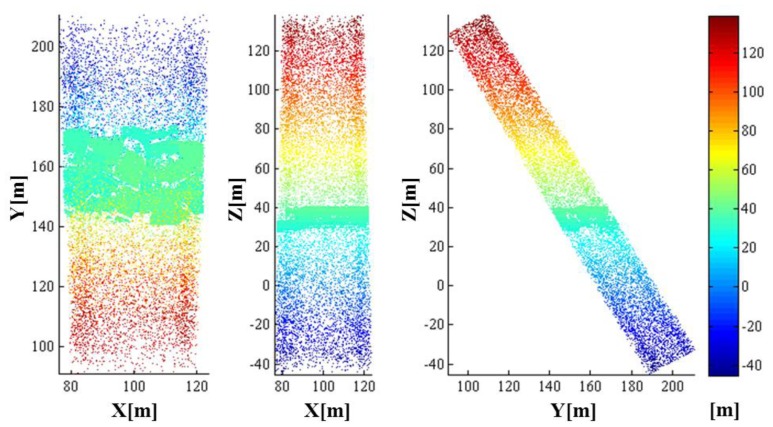
The simulated point cloud with colors encoded by height (front and side views).

**Figure 19.**
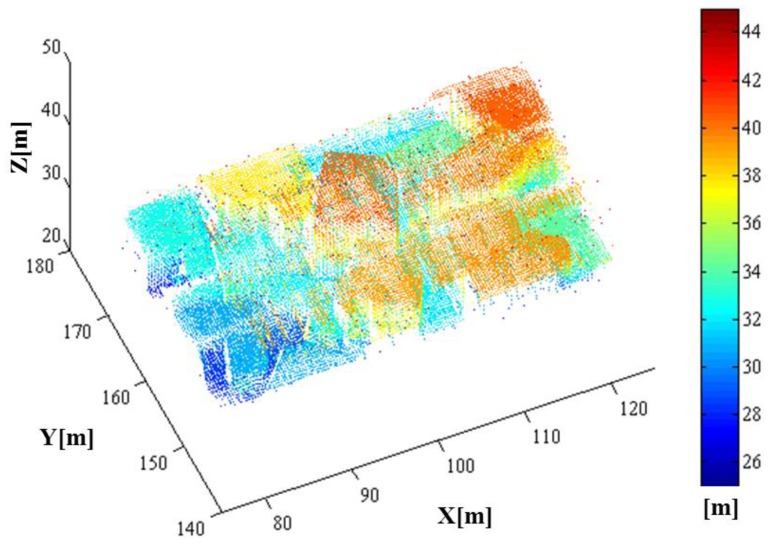
Zoom-in image of the points that are located in height of 20∼40 m to look into inlier points backscattered from target surfaces.

As a result of the whole simulation—geometry, radiometry and detection, 44,136 points were generated. The simulated point cloud generated by the entire simulation from the geometric to detection simulation is presented in Figure 18. The point density of the simulated LIDAR data was approximately 44.58 points/m^2^; the range of its x-coordinate value was 77.619∼122.230 m, and the range of its y-coordinate value was 140.620∼175.061 m. Unlike a linear mode system known to retain only a few outliers, we can confirm from the simulation results that the Geiger mode system produces significantly large number of outliers. Most outliers are caused from the dark count and the backscattered sunlight. It also includes points backscattered from target surfaces with more high density than that of outlier as shown in the middle of Figure 18 (20∼40 m height). Figure 19 represents the enlarged image of the points that are located in height of 20∼40 m to look into inlier points. The inlier point density is high enough for visual target identification.

**Figure 20.**
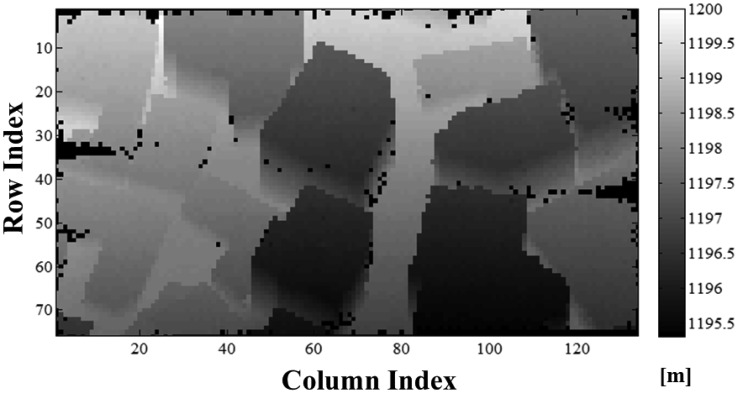
Range image (134 × 76 pixels) generated from the simulated point cloud. To generate this range image, after eliminating the outliers, we grouped the ranges according to the direction of the laser pulses with a constant angular interval.

Figure 20 shows the range image generated from the simulated point cloud. To generate this range image, outlier detection should be applied. For eliminating outliers with high ratio, we designed an adaptive median filter by analyzing the characteristic of spatial distribution of outliers [58]. The detailed algorithm will be addressed in future after being improved. After removing outliers, we grouped the ranges according to the direction of the laser pulses with a constant angular interval. The interval was determined by considering the cross-range resolution of the range image to be 0.3 m at 1 km with a look angle of 90°. Here, the sampling units of the horizontal and vertical angles were 0.0172° and 0.0171°, respectively. Then, each pixel in the range image was calculated using the average of the ranges from the corresponding laser pulses. The array size of the range image in Figure 20 was 134 × 76 pixels.

As shown in Figure 20, 558 (5.5%) of the 10,184 pixels had null values. This indicates that there are no simulated points within the view of the null pixel. The dark pixels near the edges of the image are out of the coverage range of the scanning mechanism, compared to the range image with the scanning pattern in Figure 16. The other dark pixels within the image are mainly caused by a lack of return energy. The cause of the latter dark pixels located on vertical surfaces comes from high incidence angle. In addition, the characteristics of Geiger mode APD, low incident energy due to beam profile and laser speckle (excluded in this paper) may contribute to this phenomenon. These will be addressed in future works.

**Figure 21.**
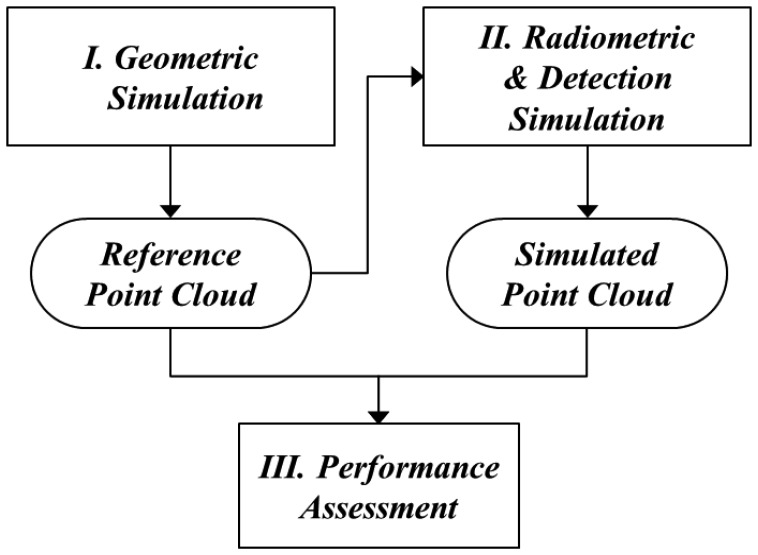
Overview of performance assessment.

The method to assess the detection performance of the LADAR system by using the simulated data with the given system parameters is as follows. As mentioned in Section 2, in general, it is difficult to identify the corresponding point pairs in two data sets of point clouds acquired by a real LADAR system. However, the pair of points between the simulated point cloud and the reference point cloud can be easily determined, because the simulated point is generated point by point from the reference data. Figure 21 describes our method of performance assessment. The results of the performance assessment based on a comparison of the simulated point cloud with the reference data set for each individual point are represented as an error matrix in Table 6.

As seen in Table 6, we categorized the reference data set into two types according to whether or not the target existed in the range gate. The former means that the pixels have to be saturated and output the range, because the target is in the range gate. The latter means that the pixels do not have to be saturated, because there is no target in the range gate. “Saturated,” the left side column in “simulation,” indicates the number of the pixels that were saturated as a result of the simulation. “Not saturated” is the number of the pixels that were not saturated. Table 7 describes the meaning of each group in the error matrix of Table 6.

**Figure 22.**
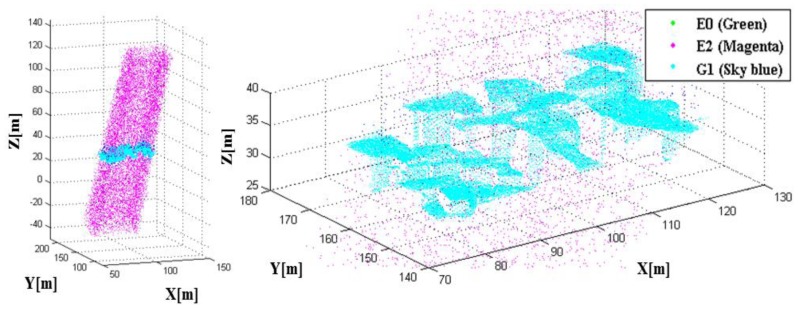
Simulated point cloud color-coded by case—E0: green, E2: magenta and G1: sky blue.

G1 and G2 are the cases where in the detection process worked correctly. E1 and E2 are cases where it did not. E1 is the dropout case in which a pixel fails to detect the return photons mainly due to a low received energy. E2 is a false alarm wherein the pixels are saturated by the noise, though there is no target in the range gate. In the Case E0, there was a target in the gate range, and the pixel was saturated similarly to G1; however, the pixels in E0 were saturated not by the laser pulse, but by the noise. In this study, we can calculate E0 by comparing the ranges between the reference and the simulated data. Figure 22 shows the point cloud color-coded into G1, E0 and E2. Based on the error matrix, we computed the indicators to assess the detection performance of a LADAR system with the given parameters in Table 8. In this study, the simulated data shows a false alarm rate of 1.76%, a dropout rate of 1.06% and an outlier proportion of 25.53%, although these indicators are not representative.

For an accurate assessment of the performance of the LADAR system with the given system parameters, multiple experimental analyses with various target models must be performed. Therefore, further studies focusing on performance assessment will be undertaken. Also, it seems to require a new method to remove outliers. There are few studies about eliminating outliers with high outlier ratio, whereas there are many studies detecting outliers from point cloud generated by linear mode LADAR with low level noise.

**Figure 23.**
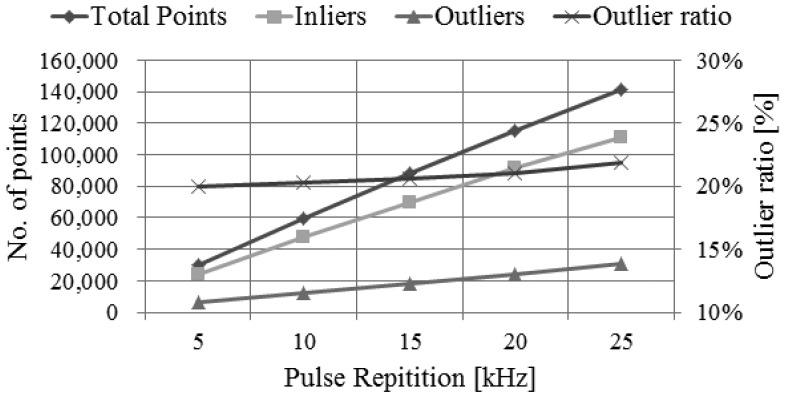
Variations of the number of total points, inliers and outliers (ratio) according to the changes of pulse repetition rate.

**Figure 24.**
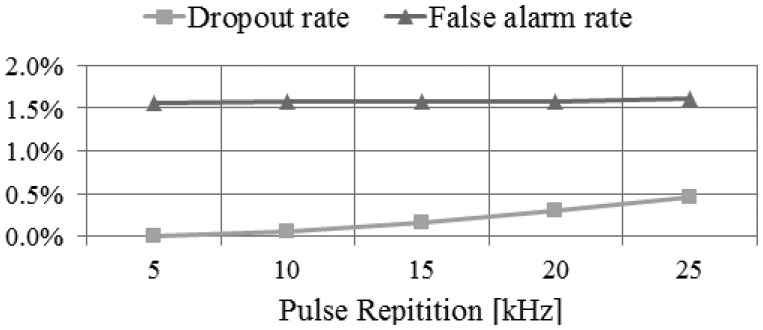
Variations of missing and false alarm rate according to the changes of pulse repetition rate.

**Figure 25.**
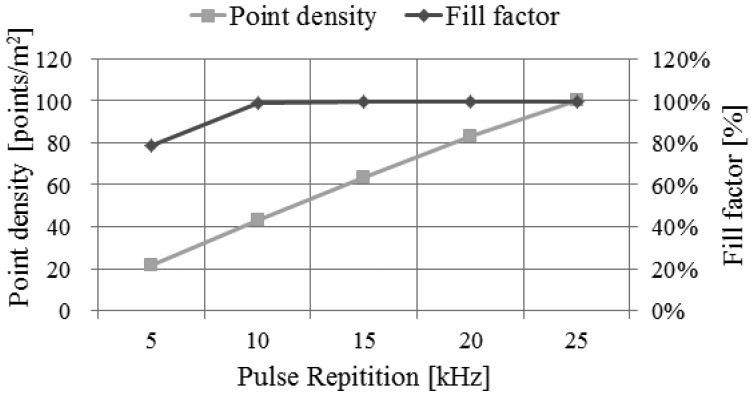
Variations of point density (inliers) and fill factor according to the changes of pulse repetition rate.

By using the performance assessment process based on simulation, we can easily analyze the impact of the main system parameters to the system performance. We can perform this analysis by evaluating the system performance derived from simulation while changing the system parameters used as the input to the simulator. For example, we performed the analysis on the impact of pulse repetition rate, as shown in Figures 23,24 and 25. We attempted to assess the performance of LADAR system by analyzing simulated data. The simulated data were generated with the system parameters in Tables 2, 3, 4 and 5 and a flat surface as a target model. So the results in Figures 23, 24 and 25 are different from those in Table 8. Figure 23 shows the variations of the number of inliers and outliers as the pulse repetition rate changes from 5 kHz to 25 kHz. The higher pulse repetition rate produces the large number of the inliers and outliers. The outlier ratio is also slightly increased. The cause can be explained by Figure 24. The false alarm rate in Figure 24 was almost uniform, because it is not related to laser energy but noises such as sunlight or dark count. However, the dropout rate is increased, because the pulse energy is decreased as in Equation (17). Figure 25 shows the performance in geometric aspect. Increasing pulse repetition rate cause higher point density of inliers owing to the increasing number of inliers as shown in Figure 23. The fill factor indicates how fully the inlier points are filled in a grid. To generate a range image from the inlier points, we divide its ground coverage into a grid with a certain ground resolution and then determine which points are corresponding to each cell. But some cells may have no point since number of inlier points may be small and (or) their distribution may be not uniform. In such cases, the fill factor can be less than 100%. As indicated in Figure 25, the pulse repetition rate should be 10 kHz at least for the maximum fill factor.

## 4. Conclusions

When developing a new LADAR system, a simulation study can be useful to assess its performance. In this paper, we propose a method for creating a simulation for a Geiger-mode imaging LADAR system and its performance assessment. The proposed simulation technique has three main parts—geometry, radiometry and detection simulations. In the geometric simulation, the sub-beam rays of the pixels are defined, and the intersection points between the rays and the target surfaces are computed using ray-tracing. Then, the radiometric simulation calculates the incident energy of the transmitted sub-beams and the noise in time domain. Finally, the detection part performs a simulation for the responses of the detector based on the probability function used by the Geiger mode detector. We confirmed that the simulated point cloud was well generated on the object surfaces and verified the range image generated using the point cloud.

Furthermore, we attempted to evaluate the detection performance. For this, we used the reference data as a result of geometric simulation. Then, we compared the simulated point cloud point by point with the reference data. The results were represented in the error matrix. The proportion of outliers in the simulated point cloud was 25.53%, and the false alarm rate of the LADAR system was approximately 1.76%. The proposed method can be applied to various applications with diverse platform and sensor systems and will be useful for such processes as system comprehension, data provision, and performance prediction.

## Figures and Tables

**Table 1.** Descriptions of the simulator modules.

**Geometry**	
Detector module	Define the sensor coordinate system and initial pixel rays
Scanning module	Compute the rotational matrix for deflection by the scanning mechanism
Movement module	Compute the position and the attitude of the vehicle at a specific time
Integration module	Transform the pixel rays from the sensor coordination system into a local coordinate system
Target module	Input the target and background model as formatted in B-rep
Ray tracing module	Search the facets intersecting with the laser pulse and compute the range and the intersection point
**Radiometry**	

Pulse module	Define the energy distribution in the temporal domain
Beam profile module	Define the energy distribution in the spatial domain
Receiver module	Calculate the incident energy using the LADAR range equation with the parameters related to the optical system efficiency
Noise module	Calculate the expected noise energy due to solar irradiance and the thermal effect of the detector's circuit
Signal module	Generate a graph representing the numbers of incident photons per time bin by pixel.
**Detection**	

Detection module	Calculate the simulated times at which the pixels are saturated based on the probabilistic model

**Table 2.** Parameters of the laser pulse (related to Equations (17) and (21)).

**Parameter**	**Variable**	**Value**
Wavelength	λ	1,560 nm
Laser mean power	*E_pulse_*	10 W
Pulse frequency	*f_pulse_*	25 kHz
Pulse width	*FWHM*	1 ns

**Table 3.** Parameters of the scanning mechanism (related to Equation (2)).

**Parameter**	**Variable**	**Lens 1**	**Lens 2**	**Lens 3**	**Lens 4**
Deflection angle	*δ_k_*	0.506 deg	0.506 deg	0.256 deg	0.256 deg
Rotational speed	*ω_k_*	45 Hz	−45 Hz	5 Hz	−5 Hz
Phase angle	*φ_k_*	0 deg	0 deg	90 deg	90 deg

**Table 4.** Parameters of the detector (related to Equations (1), (15), (16) and (22)).

**Parameter**	**Variable**	**Value**
No. of pixels		16 × 16
No. of sub-pixels		6 × 6
Pixel pitch		100 um
Dark count rate	*f_dc_*	20 kHz
Photon detection efficiency	*PDE*	0.3
Gate time		80 ns
Measurement range		200 m
System clock	*δ_t_*	1 GHz

**Table 5.** Parameters of the optics (related to Equations (1), (15) and (19)).

**Parameter**	**Variable**	**Value**
Bandpass filter transmittance	*T_BPF_*	0.5
Bandpass width	*δ_λ_*	2 nm
Transmitter optics transmittance	*T_T_*	0.8
Receiver optics transmittance	*T_R_*	0.75
Focal length	f	333 mm
Solar irradiance	*E_si_*	W/m^2^/nm

**Table 6.** Error matrix for the performance assessment of the target detection.

**Section**		**Simulation**	

		**Saturated**	**Not saturated**
Target in the range gate	Exists	32,867/451 (G1/E0)	6,791 (E1)
	Does not exist	10,818 (E2)	589,073 (G2)

**Table 7.** Descriptions of cases in Table 6.

**Case**	**Description**
G1	Target exists in gate range, and pixel is saturated by target
G2	Target does not exist in gate range, and pixel is not saturated
E0	Target exists in gate range, but pixel is saturated due to noise
E1	Target exists in gate range, but pixel is not saturated
E2	Target does not exist in gate range, but pixel is saturated by noise

**Table 8.** Indicators for the performance assessment of target detection.

**Indicator**	**Value**	**Equation**
Dropout rate	1.06%	*E*1/(*G*1 + *G*2 + *E*0 + *E*1 + *E*2)
False alarm rate	1.76%	(*E*0 + *E*2)/(*G*1 + *G*2 + *E*0 + *E*1 + *E*2)
Outlier ratio	25.53%	(*E*0 + *E*2)/(*G*1 + *E*0 + *E*2)

## References

[1] O'Brien M.E., Fouche D.G. (2005). Simulation of 3D laser radar systems. Linc. Lab. J..

[2] Vosselman G., Maas H.G. (2010). Airborne and Terrestrial Laser Scanning.

[3] Feng L., Zhiwei Y., Bo W., Qianlin D. Filtering Algorithm for LiDAR Outliers Based on Histogram and KD Tree.

[4] Meng X., Currit N., Zhao K. (2010). Ground filtering algorithms for airborne LiDAR data: A review of critical issues. Remote Sens..

[5] Sithole G., Vosselman G. (2004). Experimental comparison of filter algorithms for bare-Earth extraction from airborne laser scanning point clouds. ISPRS J. Photogramm..

[6] Sohn G., Dowman I.J. (2008). A Model-based approach for reconstructing a terrain surface from airborne lidar data. Photogramm. Rec..

[7] Lee I. (2002). Perceptual Organization of Surfaces. Ph.D. Thesis.

[8] Carlberg M. (2009). Fast Surface Reconstruction and Segmentation with Terrestrial LiDAR Range Data.

[9] Akel N.A., Filin S., Doytsher Y. (2007). Orthogonal polynomials supported by region growing segmentation for the extraction of terrain from lidar data. Photogramm. Eng. Remote Sens..

[10] Wang C.K., Lu Y.Y. (2009). Potential of ILRIS3D intensity data for planar surfaces segmentation. Sensors.

[11] Sharma M., Paige G.B., Miller S.N. (2010). DEM development from ground-based LiDAR data: A method to remove non-surface objects. Remote Sens..

[12] Haala N., Brenner C. (1999). Extraction of buildings and trees in urban environments. ISPRS J. Photogramm. Remote Sens..

[13] Vosselman G. Fusion of Laser Scanning Data, Maps, and Aerial Photographs for Building Reconstruction.

[14] Rottensteiner F. (2003). Automatic generation of high-quality building models from lidar data. IEEE Comput. Graph. Appl..

[15] Dorninger P., Pfeifer N. (2008). A comprehensive automated 3D approach for building extraction, reconstruction, and regularization from airborne laser scanning point clouds. Sensors.

[16] Sohn G., Huang X.F., Tao V. (2008). Using a binary space partitioning tree for reconstructing polyhedral building models from airborne lidar data. Photogramm. Eng. Remote Sens..

[17] Mills S.J., Castro M.P. G., Li Z.R., Cai J.H., Hayward R., Mejias L., Walker R.A. (2010). Evaluation of aerial remote sensing techniques for vegetation management in power-line corridors. IEEE Trans. Geosci. Remote Sens..

[18] Jing L., Jixian Z., Kazhong D., Zhengjun L., Qunshan S. A New Power-Line Extraction Method Based on Airborne LiDAR Point Cloud Data.

[19] Stockdon H.F., Sallenger A.H., List J.H., Holman R.A. (2002). Estimation of shoreline position and change using airborne topographic lidar data. J. Coast. Res..

[20] Hyyppä J., Kelle O., Lehikoinen M., Inkinen M. (2001). A Segmentation-based method to retrieve stem volume estimates from 3-D tree height models produced by laser scanners. IEEE Trans. Geosci. Remote Sens..

[21] Hollaus M., Wagner W., Maier B., Schadauer K. (2007). Airborne laser scanning of forest stem volume in a mountainous environment. Sensors.

[22] Flewelling J.W. (2011). Forest inventory, LIDAR, and patents. Photogramm. Eng. Remote Sens..

[23] Pal N.R., Cahoon T.C., Bezdek J.C., Pal L. (2001). A new approach to target recognition for LADAR data. IEEE Trans. Fuzzy Syst..

[24] Navarro-Serment L.E., Mertz C., Hebert M. (2010). Pedestrian detection and tracking using three-dimensional LADAR data. Int. J. Robot. Res..

[25] Grönwall C., Gustafsson F., Millnert M. (2006). Ground target recognition using rectangle estimation. IEEE Trans. Image Process..

[26] Steinvall O., Carlsson T., Gröwall C., Larsson H., Andersson P., Klasén L. (2003). Laser Based 3-D Imaging New Capabilities for Optical Sensing.

[27] Fouche D.G. (2003). Detection and False-alarm probabilities for laser radars that use geiger-mode detectors. Appl. Optics.

[28] Mallet C., Bretar F. (2009). Full-waveform topographic lidar: State-of-the-art. ISPRS J. Photogramm..

[29] Wagner W., Ullrich A., Ducic V., Melzer T., Studnicka N. (2006). Gaussian decomposition and calibration of a novel small-footprint full-waveform digitising airborne laser scanner. ISPRS J. Photogramm..

[30] Persson Å., Holmgren J., Söderman U. (2002). Detecting and measuring individual trees using an airborne laser scanner. Photogramm. Eng. Remote Sens..

[31] Zhang J., Gier A.D., Xing Y., Sohn G. (2011). Full waveform-based analysis for forest type information derivation from large footprint spaceborne lidar data. Photogramm. Eng. Remote Sens..

[32] Adams T., Beets P., Parrish C. (2012). Extracting more data from lidar in forested areas by analyzing waveform shape. Remote Sens..

[33] Aull B.F., Loomis A.H., Young D.J., Heinrichs R.M., Felton B.J., Daniels P.J., Landers D.J. (2002). Geiger-mode avalanche photodiodes for three-dimensional imaging. Linc. Lab. J..

[34] Itzler M.A., Entwistle M., Owens M., Patel K., Jiang X., Slomkowski K., Rangwala S., Zalud P.F., Senko T. (2010). Geiger-mode avalanche photodiode focal plane arrays or three-dimensional imaging LADAR. Proc. SPIE.

[35] Marino R.M., Stephens T., Hatch R.E., McLaughlin J.L., Mooney J.G., O'Brien M.E., Rowe G.S., Adams J.S., Skelly L., Knowlton R.C. (2003). A compact 3D Imaging laser radar system using geiger-mode APD arrays: System and measurements. Proc. SPIE.

[36] Milstein A.B., Jiang L.A., Luu J.X., Hines E.L., Schultz K.I. (2008). Acquisition algorithm for direct-detection ladars with geiger-mode avalanche photodiodes. Appl. Opt..

[37] Albota M.A., Aull B.F., Fouche D.G., Heinrichs R.M., Kocher D.G., Marino R.M., Mooney J.G., Newbury N.R., O'Brien M.E., Player B.E. (2002). Three-dimensional imaging laser radars with geiger-mode avalanche photodiode arrays. Linc. Lab. J..

[38] Andersson P. (2006). Long-range three-dimensional imaging using range-gated laser radar images. Opt. Eng..

[39] Laurenzis M., Christnacher F., Monnin D., Zielenski I. (2010). 3D range-gated imaging in scattering environments. Proc. SPIE.

[40] Busck J., Heiselberg H. (2004). Gated viewing and high-accuracy three-dimensional laser radar. Appl. Opt..

[41] Niclass C., Soga M., Matsubara H., Kato S., Kagami M. (2013). A 100-m range 10-Frame/s 340,x,96-pixel time-of-flight depth sensor in 0.18-μm CMOS. IEEE J. Solid State Circuits.

[42] Carlsson T., Steinvall O., Letalick D. (2001). Signature Simulation and Signal Analysis for 3-D Laser Radar.

[43] Gunderson K.S., Thomas N., Spohn T., Seiferlin K. (2004). A tradeoff investigation for the bepicolombo laser altimeter design. Proc. SPIE.

[44] Chevalier T.R., Steinvall O.K. (2009). Laser radar modeling for simulation and performance evaluation. Proc. SPIE.

[45] Graham M., Davies A., Mewett D. Simulating Foliage-Penetrating LADAR Imagery.

[46] Schenk T. (2001). Modeling and Analyzing Systematic Errors of Airborne Laser Scanners.

[47] Lohani B., Mishra R.K. Generating LIDAR Data in Laboratory: LIDAR Simulator.

[48] Kukko A., Hyyppä J. (2009). Small-footprint laser scanning simulator for system validation, error assessment, and algorithm development. Photogramm. Eng. Remote Sens..

[49] Neilsen K.D., Budge S.E., Pack R.T., Fullmer R.R., Cook T.D. (2009). Design and validation of the eyesafe ladar testbed (elt) using the ladarSIM system simulator. Proc. SPIE.

[50] Pack R.T., Saunders D., Fullmer R., Budge S. (2006). The simulation of automatic ladar sensor control during flight operations using USU LadarSIM software. Proc. SPIE.

[51] Leishman B., Budge S., Pack R. (2007). A validation procedure for a LADAR system radiometric simulation model. Proc. SPIE.

[52] Zhao M., He J., Fu Q., Xi D. (2011). Simulation and analysis about noisy range images of laser radar. Proc. SPIE.

[53] Marino R.M., Davis W.R. (2005). Jigsaw A foliage-penetrating 3D imaging laser radar system. Linc. Lab. J..

[54] Hwang S., Kim S., Lee I. (2011). Efficient geometric computation for ladar simulation. Proc. SPIE.

[55] Kim S., Min S., Kim G., Lee I., Jun C. (2009). Data simulation of an airborne LIDAR system. Proc. SPIE.

[56] Richmond R.D., Cain S.C. (2010). Direct-detection LADAR systems. SPIE Press.

[57] Jelalian A.V. (1992). Laser Radar Systems.

[58] Kim S., Lee I., Lee Y., Jo M. (2012). Outlier detection from high sensitive geiger mode imaging LIDAR data retaining a high outlier ratio. Korean J. Remote Sens..

